# scShaper: an ensemble method for fast and accurate linear trajectory inference from single-cell RNA-seq data

**DOI:** 10.1093/bioinformatics/btab831

**Published:** 2021-12-09

**Authors:** Johannes Smolander, Sini Junttila, Mikko S Venäläinen, Laura L Elo

**Affiliations:** Turku Bioscience Centre, University of Turku and Åbo Akademi University, Tykistökatu 6, 20520 Turku, Finland; Turku Bioscience Centre, University of Turku and Åbo Akademi University, Tykistökatu 6, 20520 Turku, Finland; Turku Bioscience Centre, University of Turku and Åbo Akademi University, Tykistökatu 6, 20520 Turku, Finland; Turku Bioscience Centre, University of Turku and Åbo Akademi University, Tykistökatu 6, 20520 Turku, Finland; Institute of Biomedicine, University of Turku, 20520 Turku, Finland

## Abstract

**Motivation:**

Computational models are needed to infer a representation of the cells, i.e. a trajectory, from single-cell RNA-sequencing data that model cell differentiation during a dynamic process. Although many trajectory inference methods exist, their performance varies greatly depending on the dataset and hence there is a need to establish more accurate, better generalizable methods.

**Results:**

We introduce scShaper, a new trajectory inference method that enables accurate linear trajectory inference. The ensemble approach of scShaper generates a continuous smooth pseudotime based on a set of discrete pseudotimes. We demonstrate that scShaper is able to infer accurate trajectories for a variety of trigonometric trajectories, including many for which the commonly used principal curves method fails. A comprehensive benchmarking with state-of-the-art methods revealed that scShaper achieved superior accuracy of the cell ordering and, in particular, the differentially expressed genes. Moreover, scShaper is a fast method with few hyperparameters, making it a promising alternative to the principal curves method for linear pseudotemporal ordering.

**Availability and implementation:**

scShaper is available as an R package at https://github.com/elolab/scshaper. The test data are available at https://doi.org/10.5281/zenodo.5734488.

**Supplementary information:**

Supplementary data are available at *Bioinformatics* online.

## 1 Introduction

Single-cell RNA-sequencing (scRNA-seq) is a powerful technology for studying dynamical processes of cells in tissues (Tanay and Regev, 2017). Computational models, known as trajectory inference methods, are needed to infer a representation of the cells, i.e. a trajectory, that models the real phases at which the cells are developing during the process. Trajectory inference methods have been developed to address the limitations of clustering to provide a representation that models the differentiation order of the cell types (clusters) at single-cell level, while also estimating the topology of the differentiation process. Trajectory inference methods have recently been particularly helpful in deciphering cell differentiation processes, such as human B cell maturation, innate lymphoid cell development and brown adipose tissue myeloid cell differentiation (Fiancette *et al.*, 2021; Gallerand *et al.*, 2021; King *et al.*, 2021).

A typical trajectory inference workflow includes three main steps: (i) preprocessing that includes quality control to remove lowly expressed genes and poor-quality cells, normalization and dimensionality reduction; (ii) topology inference that typically involves clustering and building a graph based on the clustering, such as a minimum spanning tree (MST); and (iii) generation of a pseudotime for each lineage of the trajectory, i.e. a set of continuous values ranging from 0 to 1 that measures the progression of the cells along the lineage (Deconinck *et al.*, 2021). While many methods have been developed for trajectory inference (Saelens *et al.*, 2019), recent works have mainly attempted to improve the topology inference (Cao *et al.*, 2019; Todorov *et al.*, 2020). The more recently developed methods include, for instance, TinGa, which uses a growing neural gas (GNG) model to build more complex trajectories like disconnected graphs, and Palantir, which models the trajectory probabilistically using a Markov process (Setty *et al.*, 2019; Todorov *et al.*, 2020). Moreover, there exists a new category of methods that use mRNA splicing information in addition to the gene expression counts, called RNA velocity methods, to improve the performance of trajectory inference (Bergen *et al.*, 2020; La Manno *et al.*, 2018). As topology inference is very challenging, users often need to try several different tools to find a suitable topology. However, in many cases a simple linear model can be sufficient, and therefore more accurate linear trajectory inference methods are still needed as well.

Linear trajectory inference is strictly limited to the linear topology that does not allow branching or cycles. For estimating the pseudotime, the most common method currently is the principal curves method, and it was recently shown that the current best linear trajectory inference methods use the principal curves method (Saelens *et al.*, 2019), including SCORPIUS and Embeddr (Campbell *et al.*, 2015; Cannoodt *et al.*, 2016). Slingshot (Street *et al.*, 2018), which was ranked as the best tree-based method in a recent comparison study, also utilizes the principal curves method (Saelens *et al.*, 2019). The principal curve is a smooth 1D curve that passes through the middle of a p-dimensional dataset (Hastie and Stuetzle, 1989). The algorithm starts from a prior curve, which is by default set to the first principal component, and then proceeds to iteratively project the samples using a smoothing function so that the samples pass through the middle of the dataset. However, it is known that the regular principal curves algorithm can perform poorly with more complex trajectories, such as spiral trajectories (Ozertem and Erdogmus, 2011), and for this reason, some scRNA-seq trajectory inference analysis methods have attempted to address this limitation using other initializers than the first principal component. SCORPIUS (Cannoodt *et al.*, 2016), which was ranked as the best linear trajectory inference method in the recent comparison study, begins by first clustering the dataset using the k-means algorithm and then infers the shortest path through the clustering and uses this as prior in the principal curves method. However, this approach raises several issues, such as how to select the number of clusters *k* and the sensitivity of the shortest path to the clustering. Moreover, it is not guaranteed that the iterative smoothing of the principal curves algorithm converges to a final curve that correlates well with the shortest path.

To address these limitations, we introduce scShaper, a new trajectory inference method that enables accurate linear trajectory inference from scRNA-seq data using an ensemble approach that combines multiple pseudotime solutions into a single more accurate ensemble solution. scShaper is based on graph theory and solves the shortest Hamiltonian path of a clustering, utilizing a greedy algorithm to permute clusterings computed using the k-means method to obtain a set of discrete pseudotimes. In contrast to other methods that rely on a single clustering, scShaper clusters the dataset multiple times using a range of different k values and combines the resulting discrete pseudotimes into a solution, which is less sensitive to the number of clusters. scShaper also excludes pseudotimes that are considered uncorrelated and aims to build the solution based on the largest subset of mutually correlating pseudotimes, effectively mitigating the instability issue of the single clustering-based approaches.

To demonstrate the benefits of scShaper, we repeated the same benchmarking as in the recent study, which compared 60 different scRNA-seq trajectory inference tools in a comprehensive manner (Saelens *et al.*, 2019). From the tools that were in the comparison, we considered four top-performing linear methods (SCORPIUS, Elpilinear, Embeddr, Component 1) and the best-performing tree method (Slingshot). We also considered one recently developed method (TinGa), which has its unique approach based on a GNG model. The results suggested that scShaper was the best method for linear trajectory inference in terms of the overall performance that measures accuracies of cell ordering and differentially expressed features. In particular, there was a considerable improvement in the accuracy of the differentially expressed features, suggesting that scShaper is able to infer trajectories that more accurately account for different cell subpopulations. Moreover, we compared scShaper with the regular principal curves method under the same preprocessing steps using the same benchmarking and found that the performance of scShaper was again statistically significantly better, suggesting that the performance boost is not only attributable to the different preprocessing steps of the trajectory inference methods, but the differences in the pseudotime inference. Conveniently, the run time of scShaper was also comparable with the other methods. Finally, we demonstrate that scShaper is also able to infer accurate trajectories for a set of different trigonometric trajectories, most of which were too complex for the regular principal curves algorithm, suggesting scShaper could become a well-generalizable alternative to the principal curves method for inferring linear paths through datasets. By replacing the principal curves algorithm with scShaper in inferring linear paths through the different lineages of non-linear trajectories (Deconinck *et al.*, 2021), this could facilitate development of more accurate non-linear trajectory inference methods.

## 2 Materials and methods

### 2.1 Linear trajectory inference algorithm of scShaper

In the following, we describe the four steps of the linear trajectory inference algorithm of scShaper that starts with preprocessed data.

**Fig. 1.**
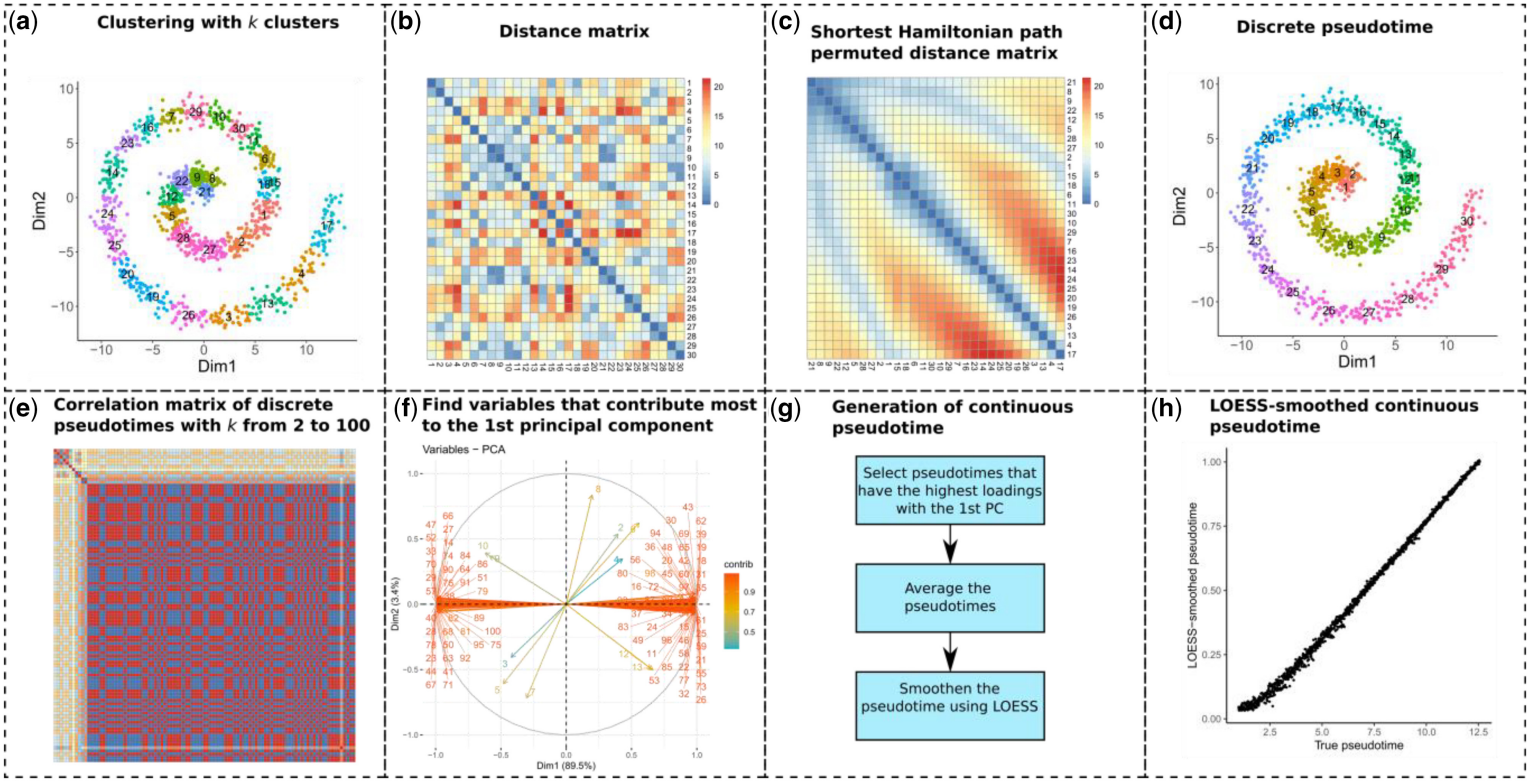
Schematic of the linear trajectory inference method of scShaper. (**a**) The data points are clustered using the *k*-means algorithm into *k* clusters. (**b**) A distance matrix is calculated between the cluster centroids. (**c**) Sort the clusters by solving the shortest Hamiltonian path problem using a greedy algorithm. (**d**) To create a discrete pseudotime, rename the original cluster labels to the permutation which rearranges the optimal permutation into ascending or descending order. (**e**) Repeat steps a–d for *k* values from 2 to 100 to obtain a set of discrete pseudotimes. The subplot visualizes a correlation matrix of the discrete pseudotimes. Red, yellow and blue colours denote high positive, low and high negative correlation, respectively. (**f**) Perform principal component analysis (PCA) to determine the largest subset of linearly correlating pseudotimes. (**g**) Schematic of the process that generates a continuous pseudotime from the discrete pseudotimes, which includes finding the pseudotimes that have the highest loadings with respect to the first principal component, averaging the selected pseudotimes and finally applying local regression (LOESS) to smooth the pseudotime. (**h**) Comparison of the ground truth and estimated continuous pseudotimes for the spiral trajectory

**1. Discrete pseudotime estimation using shortest Hamiltonian path permuted clustering.** As the basis of scShaper, we first describe an unsupervised clustering and graph theory-based algorithm that can be used to relabel clustering so that the numeric cluster labels correlate with the path through the middle of the data (Fig.  1a–d). After clustering, the labels are initially random without any dependency between the labels and neighborhoods of the clusters (Fig.  1a). Here, we refer to such a relabeled clustering that correlates with the path as the discrete pseudotime (Fig.  1d). To generate a discrete pseudotime, we need to find the permutation of the clustering that minimizes the distances between the cluster centroids of the adjacent clusters (Fig.  1b and c). From a mathematical standpoint, this is a graph theory problem, in which the clusters are the vertices and the connections between the clusters are the edges weighted by the distances between the clusters, and we seek the shortest path that visits each vertex exactly once. This kind of path, in which each vertex is visited only once, is called a Hamiltonian path, and the problem can be described as finding the shortest Hamiltonian path. However, finding the shortest Hamiltonian path is known to be NP-hard, and hence a greedy algorithm is necessary to achieve a high computational performance with large numbers of clusters. In the following, we describe a pseudocode (Algorithm 1) for our greedy algorithm, which is essentially Kruskal's algorithm for finding the minimum spanning tree (MST) of a graph. The difference is that a Hamiltonian path has slightly stricter limitations than MST, since the maximum degree of a vertex, i.e. the maximum number of adjacent edges, is two. Therefore, the problem can also be characterized as a degree-constrained MST problem. As in Kruskal’s algorithm, the greedy algorithm adds edges into the graph by starting from the shortest distance and discards connections that form cycles, but also connections that exceed the degree constraint.

After finding a solution to the shortest Hamiltonian path problem, we obtain a permutation that finds the path through the cluster centroids (Fig.  1c). Next, the discrete pseudotime (Fig.  1d) is obtained by renaming the original cluster labels (Fig.  1a) to the permutation that rearranges the optimal permutation into ascending or descending order.

Although the approach can be applied to any clustering algorithm to find a discrete pseudotime, we chose k-means due to its high computational efficiency and algorithmic simplicity. In our scShaper R package, we use the k-means implementation from the stats R package.

**2. Estimate discrete pseudotime for a range of k values**. A single discrete pseudotime that is constrained to a single number of clusters (k) is not particularly useful for scRNA-seq trajectory inference. Instead, we would like to have a continuous pseudotime that correlates with the path through the middle of the input data. Additional issues that might arise from using a single pseudotime are that the solution can be sensitive to the stochasticity of the k-means clustering and limiting the maximum degree of the graph to two means that the globally most optimal shortest path is not necessarily found using the greedy algorithm, which can consequently lead to a suboptimal discrete pseudotime. To address these limitations, we estimate the discrete pseudotime using a range of different k values (Fig.  1e), $k\in {[k}_{\min},\ldots {,k}_{\max}]$, where $k_{\min}$ and $k_{\max}$ are the lower and upper limits of the number of clusters k, respectively. An ensemble solution is created based on these clusterings, resulting in a continuous and more robust solution than a single discrete solution. Here, we always use $k_{\min}=2$ and shall constrain $k_{\max}$ in Section 3.1.

**3. Principal component analysis.** After finding a set of discrete pseudotimes for a range of different k values, scShaper uses PCA to aggregate the discrete pseudotimes into an ensemble (Fig.  1f and g). We calculate the first two principal components with prior feature standardization and determine the pseudotimes that contribute more to the first principal component than the second component based on the variable loadings. In this case, the variable loadings are the eigenvectors of PCA in a square matrix, which can be interpreted as measuring how much each variable contributes to the principal components. The objective is to find the largest set of pseudotimes that are mutually correlated and exclude pseudotimes that are uncorrelated with them. The next step is to average the selected pseudotimes to generate a crude initial pseudotime. The discrete pseudotimes are min-max scaled and ordered in the same direction using the signs of the PCA loadings, after which the pseudotime values for each cell are averaged. We found that this approach provided generally better performance than using the first principal component directly as the ensemble (see Section 3.1). To perform PCA, we use the prcomp function from the stats R package.

**4. Local regression smoothing.** As the final step, we apply local regression (LOESS) using the stats R package to smooth the crude average pseudotime, where the average pseudotime and its ranking are the response and predictor variables of the model, respectively. LOESS has two important parameters: ‘span’ that controls the degree of the smoothing (default 0.75) and ‘degree’ that determines the degree of the polynomials (default 2). We left the polynomial degree to its default, but investigated how the span parameter affected the performance in Section 3.1.

### 2.2 Preprocessing before trajectory inference

As with all scRNA-seq trajectory inference methods, several preprocessing steps are required before the actual trajectory inference with scShaper, including normalization and dimensionality reduction by feature selection, which are typically left for the user to decide. Since the benchmarking datasets (Section 2.5) were already partially preprocessed by the authors of the comparison study, including filtering outlier cells, normalization and feature selection, the only remaining step in this study was the dimensionality reduction. PCA followed by t-distributed stochastic neighbour embedding (t-SNE) (van der Maaten *et al.*, 2008) is among the most common dimensionality reduction workflows in scRNA-seq data analysis, and we therefore selected that as the default dimensionality reduction method. To perform combined PCA and t-SNE analysis, we used the Rtsne package with default parameters, including 50 principal components and the perplexity value of 30, with the exception of a 3D instead of a 2D output.

### 2.3 Additional steps after trajectory inference

After trajectory inference, the trajectory is usually visualized as a straight line through a two-dimensional visualization generated by a non-linear dimensionality reduction method, such as t-SNE, UMAP or multidimensional scaling (MDS). The user is typically also interested in investigating differential expression along the trajectory. Conveniently, the dyno R package (Saelens *et al.*, 2019) offers all these functions and supports integration with any trajectory inference method. We provide the scShaper R package together with a vignette, which integrates scShaper with commonly performed preprocessing steps (Section 2.2) and dyno to perform a full-scale trajectory inference analysis.

### 2.4 Simulation of trigonometric trajectories

We simulated several trigonometric trajectories, which are summarized in Supplementary Table S1, to constrain the parameters of scShaper (Section 3.1), including 2D spirals, quadratically growing 2D spirals, 3D spirals, quadratically growing 3D spirals and sine waves. Using the trigonometric trajectory functions and a sequence of *t* values with increments of 0.01, we, generated datasets with 1157 to 1785 samples. In addition, we simulated several trajectories with each function by adding a varying amount of Gaussian noise into the variables by adjusting the standard deviation of the Gaussian distribution from 0.05 to 0.95. After adding the noise, the exact ground truth pseudotime becomes unknown, but the *t* variable still provides a good approximation of the ground truth. The analysis of these trajectories provides a means to constrain the parameters of scShaper in a manner that is not biased towards the scRNA-seq benchmarking.

### 2.5 Benchmarking

To benchmark scShaper for trajectory inference of scRNA-seq data against current state-of-the-art methods, we used a framework from a recent comparison study (Saelens *et al.*, 2019), which benchmarked over 60 different methods using a wide array of different datasets, both simulated and real, and 17 different performance metrics.

The benchmarking data included in total 69 linear datasets, of which 39 were real and 30 were simulated datasets. The real datasets included gold and silver datasets. For the gold datasets, the discrete pseudotime is known, i.e. the different cell types and the order in which they differentiate. The silver standard datasets include continuous pseudotimes, which were inferred using scRNA-seq trajectory inference methods by the authors of the original studies. The simulated datasets were simulated using four different simulators: dyntoy (Saelens *et al.*, 2019), dyngen (Saelens *et al.*, 2019), PROSSTT (Papadopoulos *et al.*, 2019) and Splatter (Zappia *et al.*, 2017) and provided the most accurate ground truth.

Of the 12 linear trajectory inference methods that were benchmarked in the comparison study (Saelens *et al.*, 2019), we selected four linear methods for our comparisons. SCORPIUS (Cannoodt *et al.*, 2016), Component 1 and Embeddr (Campbell *et al.*, 2015) were the three most highly ranked methods, whereas the fourth selected method, ElPiGraph Linear (Elpilinear) (Albergante *et al.*, 2020), is the only method that uses some form of ensemble learning, although much different compared with the approach of scShaper. SCORPIUS and Embeddr use the principal curves method in the actual pseudotemporal ordering step, but they differ in their approach to dimensionality reduction: SCORPIUS uses MDS, whereas Embeddr uses Laplacian eigenmaps. Component 1 is the simplest algorithm, which only computes the first principal component and uses it directly as the pseudotime. ElPiGraph Linear is an extension of ElPiGraph, which is limited to linear trajectories. ElPiGraph has its own considerably more unique approach based on elastic principal graphs, which resembles the principal curves method, but also allows branching to model more complex topologies. ElPiGraph also utilizes ensemble learning to build a more accurate consensus solution based on multiple principal graphs, making it hence a good method to compare with scShaper.

We also considered two non-linear trajectory inference methods: Slingshot and TinGa (Street *et al.*, 2018; Todorov *et al.*, 2020). Slingshot can infer tree-shaped trajectories, whereas TinGa can also infer disconnected trajectories and cycles. Therefore, both methods can also find linear trajectories, and the shape of the trajectory is estimated automatically by both methods. Slingshot was ranked as the best tree-based trajectory inference method in the comparison study (Saelens *et al.*, 2019), and it has become widely used since then. It estimates a tree-shaped topology using MST, which is fit to a Gaussian mixture model clustering, followed by simultaneous fitting of multiple principal curves for the different lineages of the tree. TinGa is a more recently introduced method based on a growing neural gas model (Fritzke, 1995), for which the authors showed improved generalizability compared to Slingshot.

To measure the overall performance of the methods, we followed the dynbenchmark benchmarking framework of the previous comparison study, which used the geometric mean of four metrics that measure (i) the accuracy of cell ordering (cordist), (ii) the accuracy of the differentially expressed features (wcor), (iii) the accuracy of the topology inference (HIM) and (iv) the accuracy of the branching points (F1_branches), of which the last two are always constant with value 1 (perfect) in linear trajectory inference. Therefore, in linear trajectory inference the overall performance was determined by cor_dist and wcor so that low scores in either metric were penalized by the geometric mean. Here, cordist quantifies the similarity between the known and predicted trajectories in terms of correlation of pairwise distances between the two trajectories, and wcor quantifies the agreement between the differentially expressed features obtained using the known and the predicted trajectories. The results for all the 17 performance metrics are provided in Supplementary Figures S4–S6, and the metrics are briefly described in Supplementary Table S2. More detailed descriptions of the measures are given in the documentation of dynbenchmark (https://github.com/dynverse/dynbenchmark).

To determine whether the observed performance boost for scShaper was statistically significant, we used the Wilcoxon signed-rank test from the stats R package. The related benchmarking code is available online at https://github.com/elolab/scShaper-benchmarking.

### 2.6 Software and hardware for measuring run time

The run times of the five benchmarked methods were measured on a laptop with Ubuntu 16.04 LTS operating system, 2-core 2.30 GHz Intel(R) Core (TM) i5-6200U processor and 8 GB DDR4 of RAM. As data, we used three datasets with 1000, 5000 and 10000 cells, each with 1000 features, simulated using the dyntoy tool.

## 3 Results

### 3.1 Constraining parameters of scShaper

To constrain the parameters of scShaper, we considered 50 different datasets that were simulated from five different trigonometric functions with varying levels of Gaussian noise added into the variables (Section 2.4). We ran scShaper with different parameter configurations and calculated the root mean squared error (RMSE) and the Pearson correlation coefficient between the approximative ground truth and estimated pseudotime for each dataset.

**Fig. 2.**
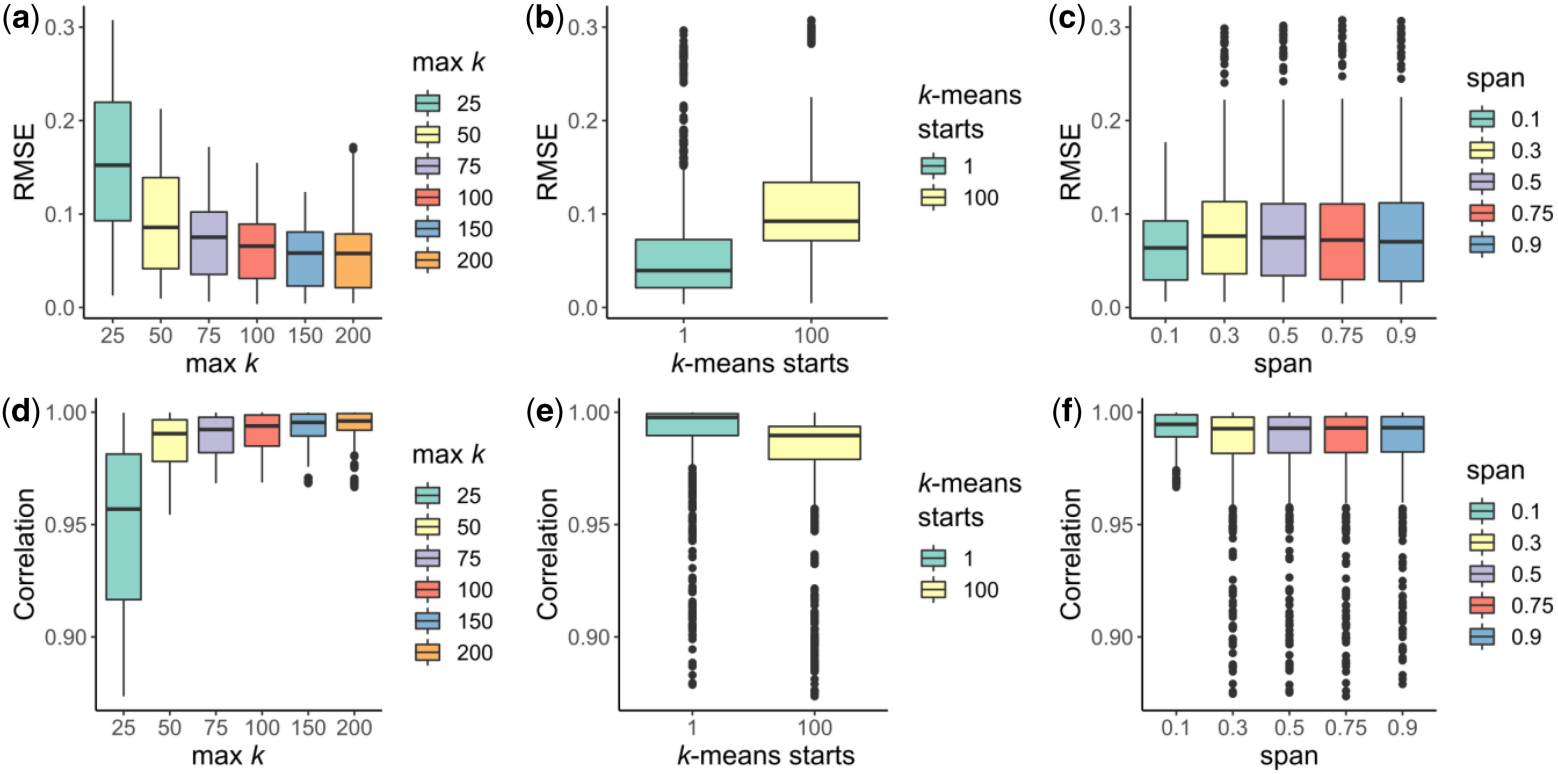
Analysis of simulated trigonometric trajectories to constrain the three hyperparameters of scShaper. (**a, d**) The maximum number of clusters for which the discrete pseudotime was calculated using the *k*-means algorithm, the minimum being 2. (**b, e**) The number of k-means initializations. (**c, f**) The span parameter that controls the degree of smoothing in Local Polynomial Regression Fitting (LOESS). *y*-axis in the subplots a–c and d–f signify the root mean squared error (RMSE) and the Pearson correlation coefficient, respectively, calculated for all the datasets **(**Section 2.4) between the ground truth and inferred pseudotimes

The results suggest that the two *k*-means clustering related parameters, the maximum number of clusters (Figs.  2a and d) and the number of random sets in *k*-means (Figs.  2b and e) had significant effects on the performance of scShaper. scShaper performed better with a higher maximum *k* value, which is intuitive considering this adds more information into the ensemble set. However, as increasing the upper limit of the *k*-value also increases the run time (Supplementary Fig. S1), we concluded that *k = 100* would be a good trade-off between the run time and the performance. The number of *k*-means initializations had an inverse effect on the performance, where one initialization outperformed hundred initializations. This occurs due to the fact that increasing the number of initializations increases the robustness of the clustering. However, our algorithm benefits from less robust clustering, because this way we obtain more dissimilar discrete pseudotimes, which subsequently provides more information about the relative positions of the cells in the trajectory. Since using a smaller value also helps to minimize the run time of scShaper, we set the default value of the number of *k*-means initializations to 1.

The third and final parameter, span, controls the degree of smoothing in LOESS, where a higher value smooths the curve more. Unlike with the two *k*-means related parameters, the differences between the performances obtained using different span values were relatively small (Fig.  2c and f). Because the analysis suggested that a lower degree of smoothing was, in general, better, we set the default value of span to 0.1.

Since the modified Kruskal’s algorithm for finding the shortest Hamiltonian path is greedy, it is not necessarily able to find the globally most optimal solution, unlike the Kruskal’s algorithm for MSTs. This makes the algorithm somewhat sensitive to the input sequence of the clusters (vertices). Therefore, we investigated robustness of the ensemble method using 1000 different random initializations so that the random shuffling of the cells before analysis and the clustering results of *k*-means vary. We used the default parameters selected above and a 3D spiral as the dataset. The correlation values visualized in Supplementary Figure S2 had only minor variation, showing that scShaper performs robustly.

In addition, we investigated two different approaches for aggregating the discrete pseudotimes into the ensemble pseudotime: (i) by comparing the PCA loadings of the first two principal components to select features that correlate more with the first principal component and (ii) using the first principal component directly. The feature selection approach provided better performance in 86% of the datasets when measured by the Pearson correlation (Supplementary Fig. S3).

### 3.2 Benchmarking using scRNA-seq data

**Fig. 3.**
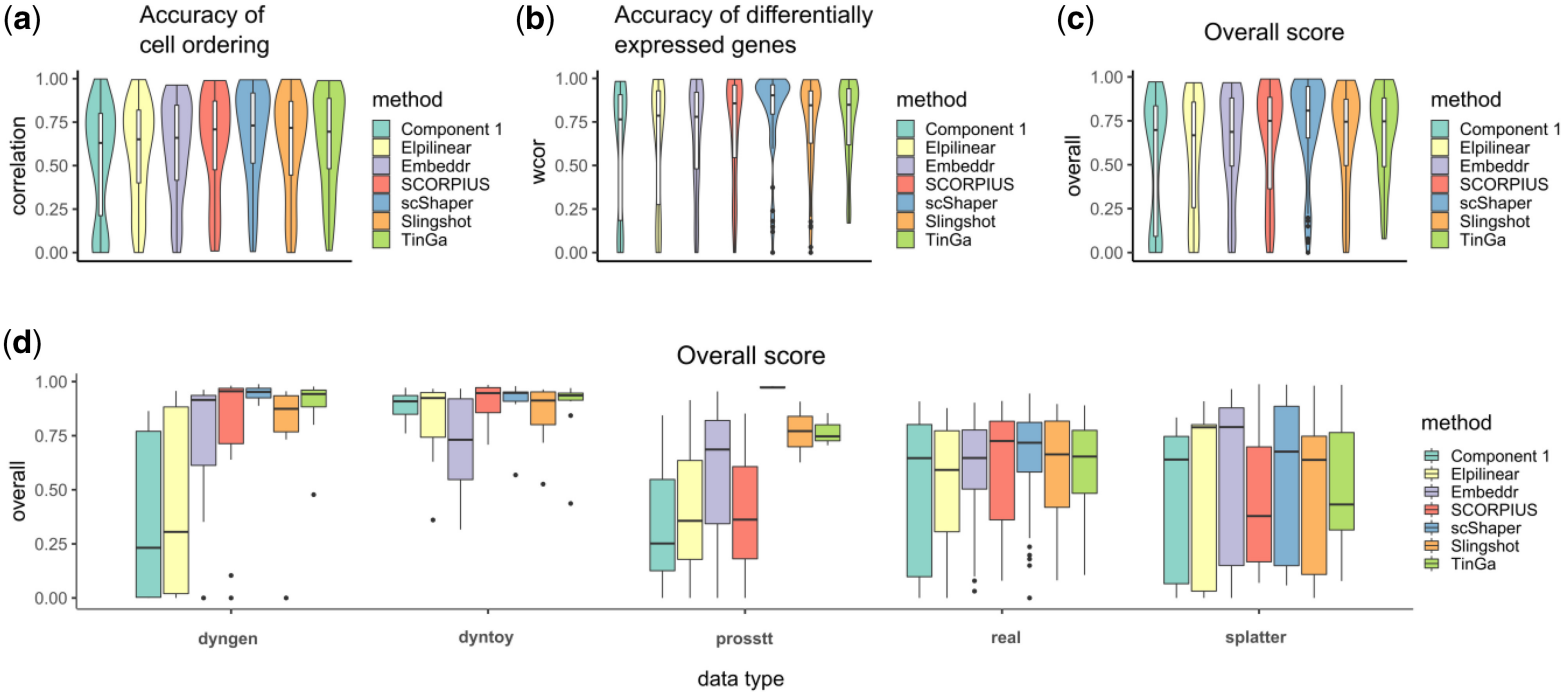
Benchmarking results for scRNA-seq data. (**a**) Accuracy of the cell ordering (cordist). (**b**) Accuracy of the differentially expressed features (wcor). (**c**) The overall score based on the geometric mean of the two previous metrics. (**d**) The overall scores grouped by data type, where dyngen, dyntoy, prosstt and splatter are simulators and real denotes real data

We benchmarked scShaper against four linear scRNA-seq trajectory inference methods, SCORPIUS, Component 1, Embeddr, ElPiGraph Linear (Elpilinear) and two non-linear methods, Slingshot and TinGa, using the current best practices (Section 2.5). Figure  3 visualizes the main results of the benchmarking across all the real and simulated datasets. The results suggested that the cell ordering (cordist) of scShaper was at least as accurate as that of the four other methods (Fig.  3a; P<0.05 compared with Component 1, Elpilinear, TinGa and SCORPIUS; P=0.1 compared to Embeddr and Slingshot). The second correlation metric (wcor), measuring the accuracy of the differentially expressed features, was consistently higher for scShaper than for the other methods (Fig.  3b; P<0.01 compared with all other methods). As in the original comparison study, we calculated the geometric mean of these two metrics to determine the overall score (Fig.  3c). The results suggested again that scShaper was the best method (P<0.05 compared with all other methods).

Next, we grouped the overall scores by the type of the data, i.e. which simulator was used or if the data were real. According to these results (Fig.  3d), scShaper showed superior performance for three of the simulators (dyngen, dyntoy, prosstt) and also yielded the highest average performance for the real datasets. For dyngen, dyntoy and real datasets, the margins between the medians of SCORPIUS and scShaper were small, but compared to SCORPIUS, scShaper had noticeably fewer datasets with low performance. The datasets simulated using splatter were a clear exception in the sense that there were several datasets, which none of the methods were able to model accurately. The performance of scShaper for splatter datasets was moderate, whereas SCORPIUS and TinGa exhibited inferior performance. The two non-linear methods (Slingshot and TinGa) did not outperform scShaper with any of the data types, but their performance was generally better or similar compared to the other linear methods.

The whole comparison using the dynbenchmark framework included in total 17 benchmarking metrics, the remaining of which are visualized in Supplementary Figures S4–S6. Notably, investigation of the Hamming-Ipsen-Mikhailov (HIM) similarity of the topologies and the branch assignments (F1_branches) indicated that the non-linear methods failed to predict the correct topology and the branch assignment of the cells was inaccurate for many of the datasets (Supplementary Figs. S4 and S5). TinGa was generally more affected by this issue than Slingshot. Most of the metrics had similar rankings between the methods and different types of simulated or real data as in the comparison of the overall scores (Fig.  3). However, the F1_milestones metric that measures the accuracy of milestone assignment using clustering comparison (in this case, the trajectory has two milestones: the start and end points) suggested scShaper as the best method also for the Splatter simulated data (Supplementary Fig. S4), although it was moderate when measured by the accuracy of cell ordering (cordist). This suggests that scShaper performs significantly better than the other methods when transforming the trajectories to discrete pseudotimes. The trajectories of scShaper were also better predictable using a random forest regression model than a linear regression model when measured by the normalized mean squared error (rf_nmse; Supplementary Fig. S6).

### 3.3 Comparison of the principal curves method and scShaper

**Fig. 4.**
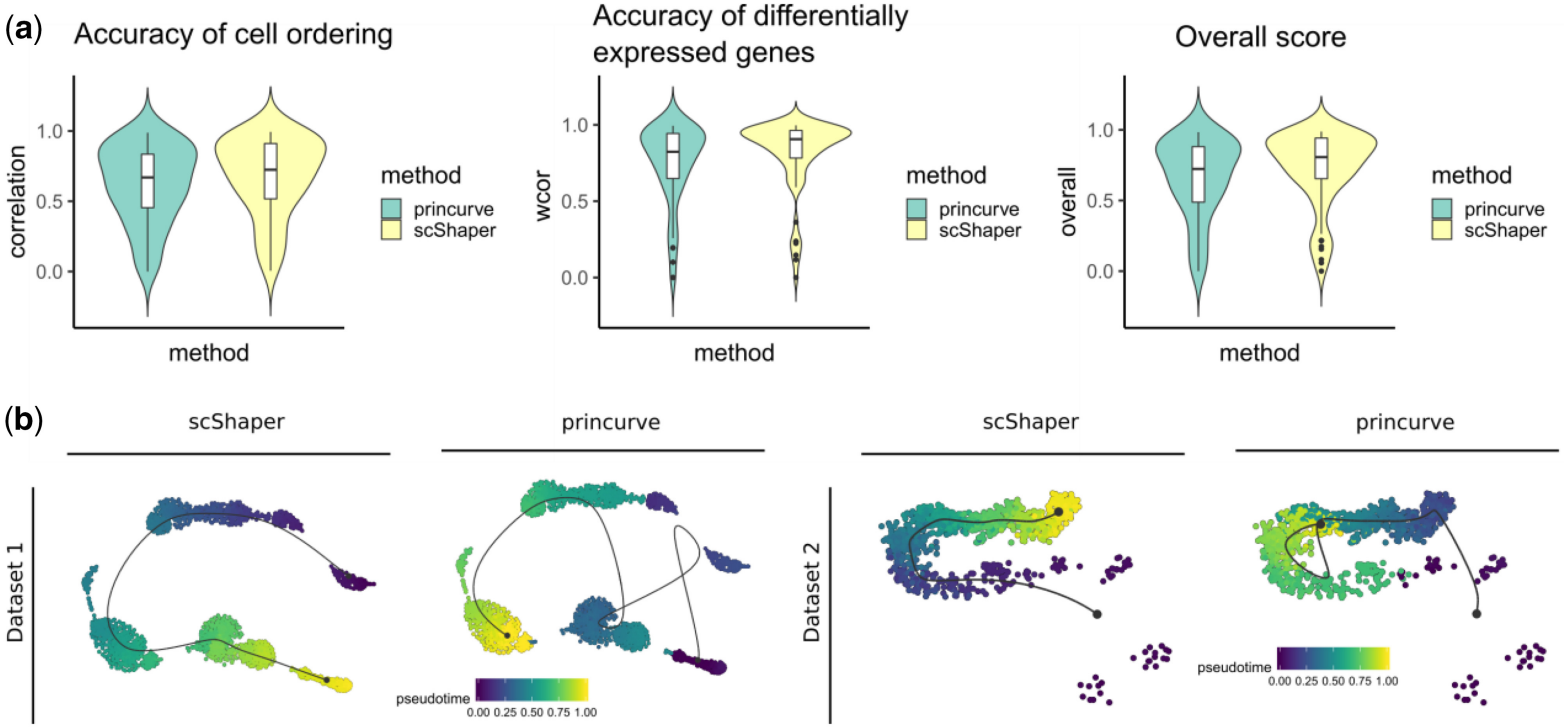
Comparison of the principal curve method (princurve) and scShaper for linear trajectory inference of scRNA-seq data. (**a**) The same benchmarking as in Section 3.2. (**b**) Trajectory visualizations of two simulated datasets, prosstt_linear_3 and prosstt_linear_6, from the comparison study. The colouring in each plot denotes the estimated pseudotime by the method. In all visualizations the trajectory line estimated using the dynplot R package is an approximation of the estimated pseudotime, and therefore the trajectory line bypasses some of the small subpopulations with fewer cells. In all the analyses we used the same dimensionality reduction workflow (Section 2.2) with both methods

The principal curves algorithm is a widely used method in scRNA-seq trajectory inference and it is commonly applied at the final phase of trajectory inference to generate a continuous pseudotime. Since the performance differences observed in the benchmarking could be partially attributable to the different preprocessing steps, we investigated how the performances of principal curve method and scShaper compare when using the same preprocessing steps (Section 2.2). In this comparison, we used the implementation of the principal curves algorithm from the princurve R package, which is used by several popular trajectory inference methods, such as Slingshot and SCORPIUS. We performed the same benchmarking as in Section 3.2 and the results suggested (Fig.  4a) that scShaper was better in terms of the overall score, as well as in terms of the accuracies of the cell ordering and the differentially expressed features (P< 0.05).

Next, we considered two datasets from the earlier benchmarking (Fig.  3) that were particularly challenging (prosstt_linear_3 and prosstt_linear_6) and visualized the estimated trajectories using the dynplot R package (Fig.  4b). For both datasets scShaper was able to generate trajectories that had almost perfect cell ordering accuracies (Fig.  3d), whereas the trajectories predicted by the principal curve method were highly inaccurate.

Finally, we investigated how accurately the two methods were able to predict the path through the middle of each of the simulated trigonometric trajectories, which included 2D and 3D spirals and the sine wave (Section 2.4). While both methods were able to accurately predict the path through the sine wave, only scShaper predicted accurately the ordering of the spiral trajectories (Supplementary Fig. S7).

### 3.4 Run time

**Fig. 5.**
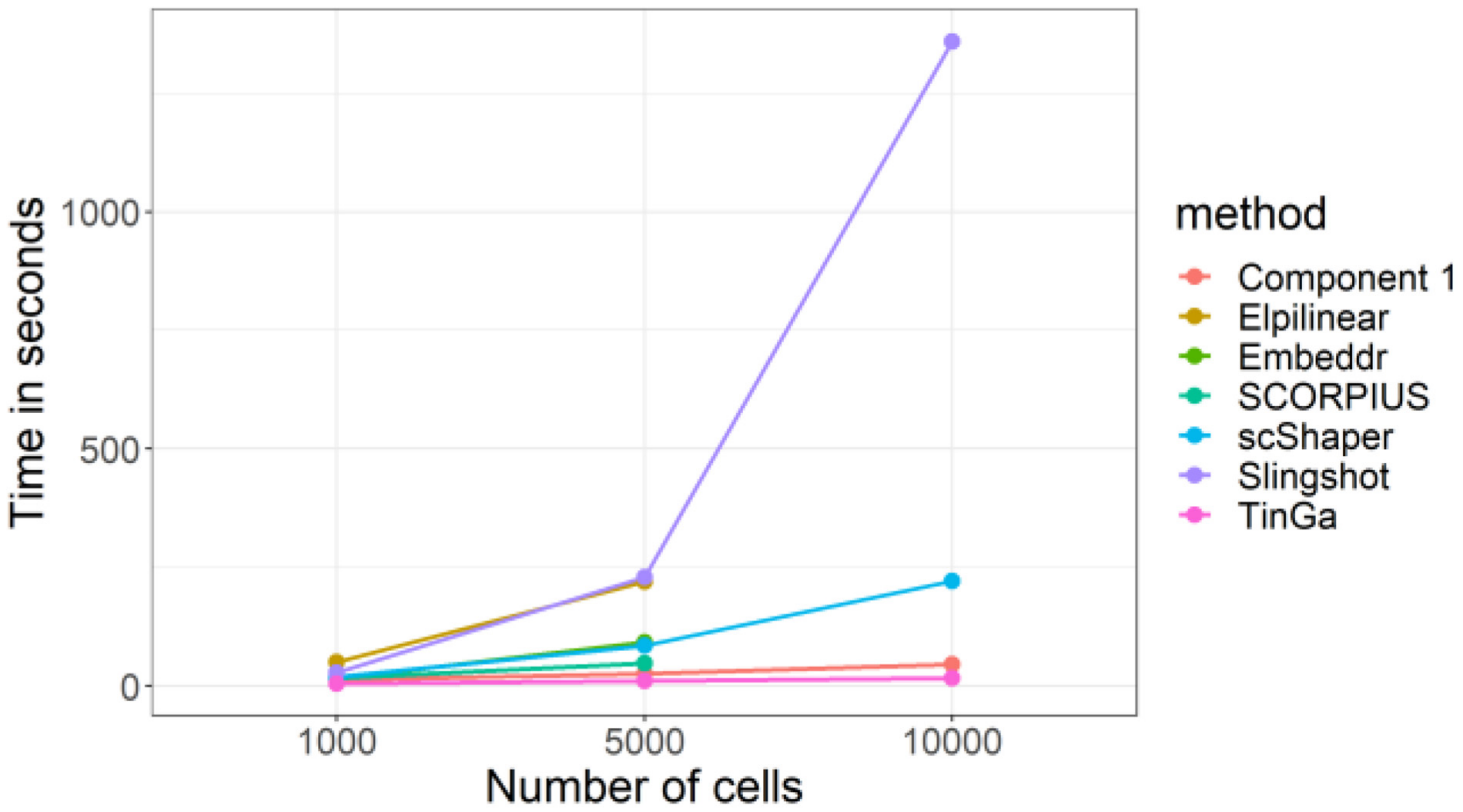
Run time comparison for the five benchmarked methods. With 10 000 cells, SCORPIUS, Embeddr and Elpilinear were unable to complete the analysis without memory failure on a laptop with 8 GB of RAM

The run time of scShaper mostly depends on three factors. First, it depends on the range of the *k* values used to estimate the discrete pseudotime. Our analysis shows (Supplementary Fig. S1) that the fast Rcpp implementation of the greedy algorithm in the scShaper R package can permute 99 distance matrices with *k* values from 2 to 100 in roughly 2.5 s. When the upper limit of the *k* values was increased to 200, the average run time was almost 1 minute. Second, the run time depends on the size of the dataset, i.e. how many features and cells it contains. Figure  5 visualizes the results of a run time comparison for the five benchmarked methods, where the dyntoy tool was used to simulate 1000, 5000 and 10 000 cells with 1000 features. The run times of the seven methods were on a largely similar scale, being at most a few minutes. For scShaper, the analysis took 19 s and 120 s with 1000 and 5000 cells, respectively. However, some of the methods had a relatively high memory requirement. Embeddr, SCORPIUS and Elpilinear had a memory failure with 10 000 cells on a laptop with 8 GB of RAM. For the largest dataset with 10 000 cells, the run time of scShaper was about 4 min, which is considerably faster than Slingshot, but slower than TinGa and Component 1. Finally, the run time also depends on the efficiency of the dimensionality reduction. Dimensionality reduction using PCA and *t*-SNE was clearly the slowest step of the scShaper’s workflow with all three datasets.

## 4 Discussion

The current state-of-the-art trajectory inference methods, such as SCORPIUS and Slingshot, perform a single clustering, which is then used as the basis for inferring the topology of the trajectory using greedy graph theory algorithms, such as Kruskal’s algorithm. However, there are several issues with this approach that can deteriorate the performance. Greedy algorithms can sometimes only find a local optimum, which is not necessarily the most optimal solution that accurately models the path through the real process. The clustering algorithms themselves, such as k-means, are stochastic, and the estimation of the number of clusters is known to be very challenging (Kiselev *et al.*, 2017). All these issues make trajectory modelling a challenging task, which can be sensitive to small fluctuations in the input data and the model parameters. In addition, the principal curves algorithm, which is commonly used in the final step to smooth each lineage of the trajectory, can significantly alter the path given as prior information and oversimplify the trajectory by bypassing many important milestones (Todorov *et al.*, 2020).

In this article, we introduced scShaper, a novel linear trajectory inference method that utilizes an ensemble learning approach that combines multiple discrete pseudotimes derived from multiple clusterings. Ensemble methods are machine learning methods that combine multiple solutions to build a model that performs better than the individual models. They have already been widely adapted in scRNA-seq data analysis, especially in consensus clustering and cell-type classification (Lieberman *et al.*, 2018; Smolander *et al.*, 2020). However, so far there have been few attempts to apply the principle of ensemble learning to trajectory inference, although the same challenges are present there as well (Albergante *et al.*, 2020).

The ensemble approach of scShaper combines multiple discrete pseudotimes derived from multiple clusterings using different numbers of clusters to build a more accurate trajectory. Instead of specifying a single number of clusters, the user only needs to select a range of cluster numbers (default from 2 to 100). For each clustering, we estimate a discrete pseudotime using a greedy algorithm that is a modification of Kruskal’s algorithm for finding MSTs. scShaper applies PCA to find subsets of mutually correlating pseudotimes, and an ensemble solution is built based on the largest subset of mutually correlating pseudotimes. Finally, we use LOESS to perform smoothing and generate the final continuous smooth pseudotime. scShaper mitigates the shortcomings of the current state-of-the-art methods, e.g. SCORPIUS, that use a single clustering as the basis for building the trajectory, making it less sensitive to the stochasticity of the clustering and the greediness of the graph theory algorithms that find the shortest path through the clustering.

We repeated the same comprehensive comparison of scRNA-seq trajectory inference methods that was recently published, which involved a wide array of different performance metrics and datasets of real and simulated origin (Saelens *et al.*, 2019). The comparison showed that scShaper achieved superior accuracy for differentially expressed genes, while still maintaining accurate cell ordering. Indeed, the overall performance score of scShaper was best for most of the simulators and the real data. This suggests that scShaper is able to maintain a high cell ordering accuracy and model more accurately subpopulations that are bypassed by other methods. The performance of scShaper was also better than that of the two non-linear trajectory inference methods (TinGa, Slingshot), suggesting that methods that have been designed for specific topologies can outperform methods that have been designed to work with arbitrary topologies. Therefore, the generalizability of trajectory inference methods needs to be still improved.

Another significant part of this work included comparing the principal curves method and the pseudotemporal ordering algorithm of scShaper. Since the principal curves algorithm has become so widely utilized in scRNA-seq trajectory inference, we compared it with scShaper under the same preprocessing steps. The results showed that scShaper outperformed the principal curves algorithm in terms of all three metrics that were used in the evaluation. Moreover, scShaper achieved excellent accuracy for different trigonometric trajectories, including spiral functions, which the principal curves method failed to accurately model. Since many of the benchmarked methods utilize the principal curves algorithm, this strongly suggests that the performance boost of scShaper is not attributable to the differences in the dimensionality reduction, but to the differences in the pseudotemporal ordering.

The benefits of scShaper compared with the principal curves algorithm suggest that it can facilitate development of more accurate non-linear trajectory inference methods. By replacing the principal curves algorithm with scShaper in the lineage smoothing step (Deconinck *et al.*, 2021), this could improve the accuracy of differentially expressed genes compared with methods like Slingshot. This would require a simple modification to the aggregation of scShaper to select the discrete pseudotimes that have a high correlation with the lineages. Another issue would be how to handle the branching points, which could be done in a similar way as Slingshot constructs average curves from multiple principal curves.

scShaper is a relatively simple algorithm with few hyperparameters. The most important parameter is the range of different *k* values, i.e. the number of clusters in *k*-means clustering, for which the discrete pseudotimes are estimated. Adjusting the range may be necessary, if the trajectories significantly exceed the complexity of the trajectories considered in this work. This can for example happen when we add considerably more rounds to the spiral trajectories (Section 2.4). The run time of scShaper largely depends on the range of *k* values (Section 2.6). For a set of clusterings, where *k* ranges from 2 to 100, the shortest path estimation lasted only 2 s, but when the upper limit was increased to 200, the analysis was noticeably longer (∼ 1 min). It should also be noted that the algorithm cannot automatically infer the correct direction of the trajectory, which is a common limitation among trajectory inference methods. This requires the user to define the starting point, for example, based on gene markers. The flipping can be easily performed by subtracting the pseudotime values element-wise from 1.

To conclude, scShaper is a new fast and accurate method for linear trajectory inference from single-cell RNA-seq data. Our comparison showed that it outperformed current state-of-the-art methods (SCORPIUS, Embeddr, Slingshot) that utilize the principal curves method and also three other accurate methods (ElPiGraph Linear, Component 1, TinGa). We observed a particularly large improvement in the accuracy of the differentially expressed genes, which we hypothesized to be related to the over-smoothing behaviour of the principal curves algorithm, making the principal curves algorithm to bypass some of the trajectory milestones. Further analysis suggested that the performance boost of scShaper is indeed not attributable to the dimensionality reduction, but the ensemble approach for generating the continuous pseudotime. scShaper can be downloaded as a user-friendly R package at https://github.com/elolab/scshaper.

## Supplementary Material

btab831_Supplementary_Data
