# Supplementary material for: scShaper: an ensemble method for fast and accurate linear trajectory inference from single-cell RNA-seq data
Source: Bioinformatics. 2021 Dec 9;38(5):1328–35. doi: 10.1093/bioinformatics/btab831 (PMC8825760; doi:10.1093/bioinformatics/btab831)
Supplement: btab831_Supplementary_Data [file btab831_supplementary_data.pdf]

Supplementary Information

Supplementary Figure 1

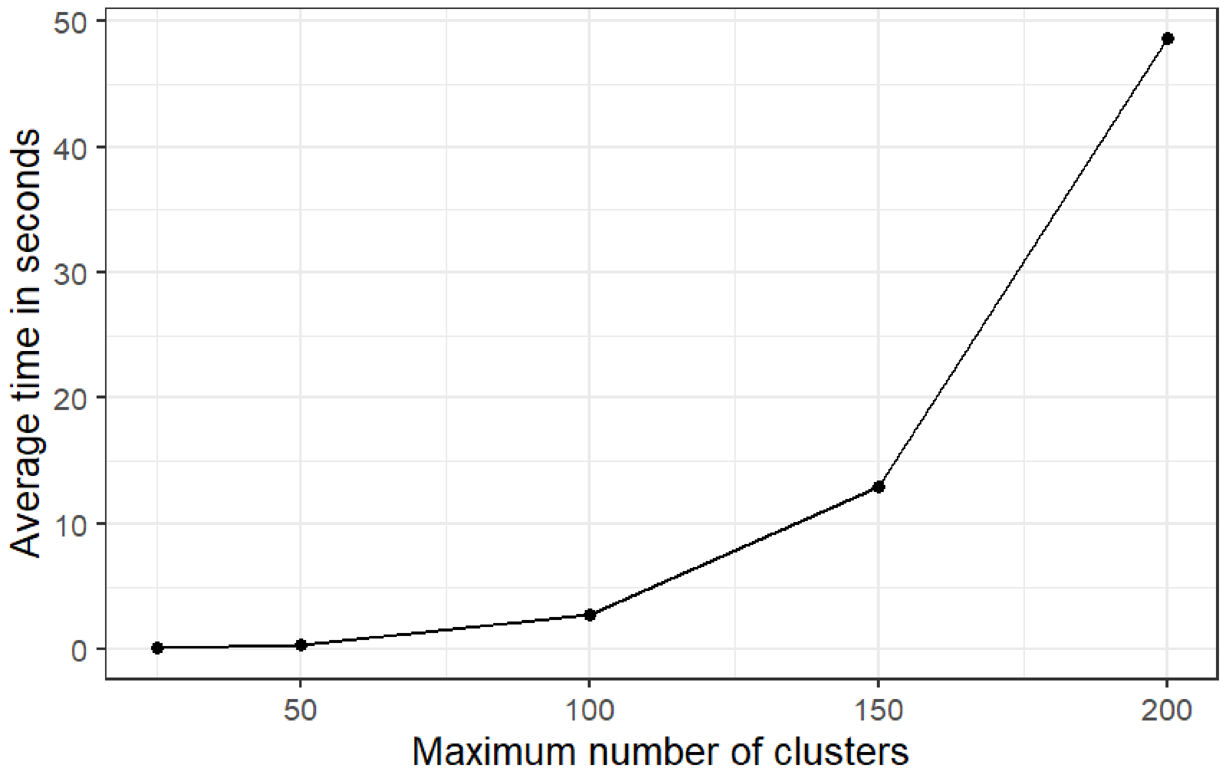

Supplementary Figure 1. Average run time in seconds of 100 repetitions of $k_{max} - 1$ greedy algorithm runs that solve the shortest Hamiltonian path problem for distance matrices with $k \in \{2,..., k_{max}\}$ clusters, where k_{max} is the maximum number of clusters. The greedy algorithm is implemented in Rcpp in the scShaper R package.

Supplementary Figure 2

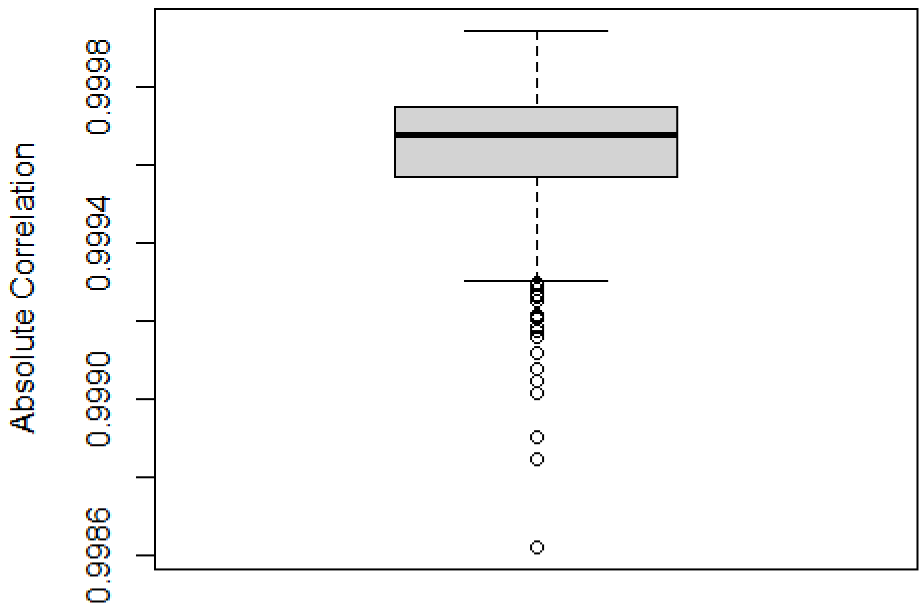

Supplementary Figure 2. Absolute values of Pearson correlation coefficients from 1000 scShaper runs to study robustness of scShaper. The runs were initialized with different random number generation seeds so that the random shuffling of cells before analysis and the results of the *k*-means clustering varied between the runs. The data set was a 3D spiral trajectory (see **Supplementary Table 1**), which is the same trajectory that is available on Github as an example (https://github.com/elolab/scshaper).The noise added to the coordinates was randomly sampled from a Gaussian distribution with standard deviation of 0.05.

Supplementary Figure 3

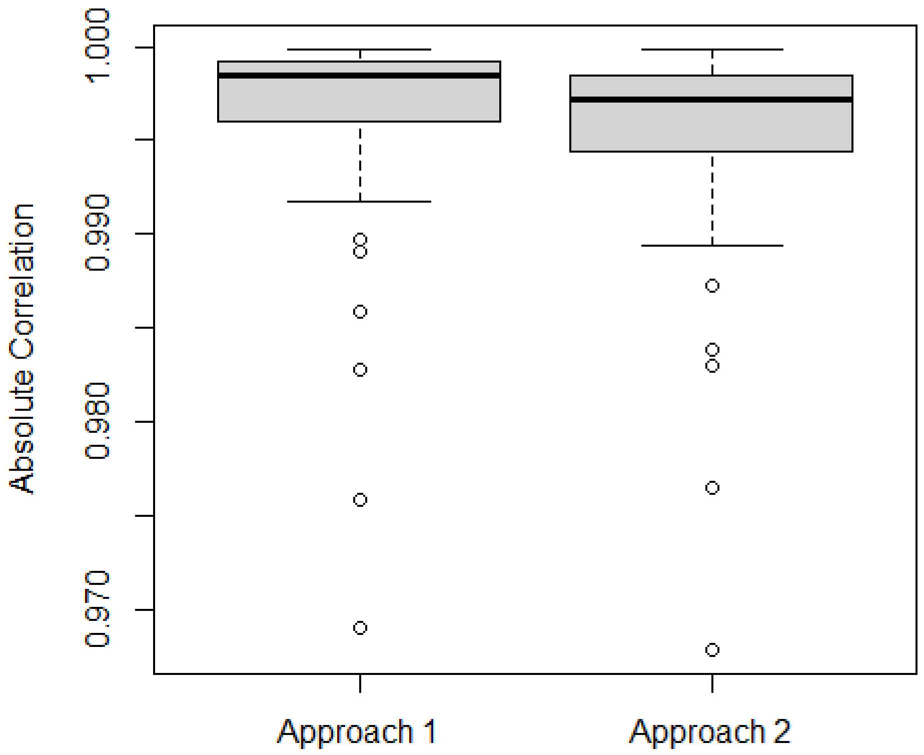

Supplementary Figure 3. Comparison of two approaches for pseudotime aggregation. Approach 1 denotes the aggregation approach of scShaper that selects the features that have a higher contribution to the 1st principal component than the 2nd principal component (see **Section 2.1**). Approach 2 uses the 1st principal component directly as the ensemble. In addition, the aggregated pseudotimes of both approaches were smoothed using LOESS to obtain the final continuous pseudotime. Each data point signifies the absolute value of the Pearson's correlation coefficient for one of the 50 trigonometric trajectories (see **Section 2.4** and **Supplementary Table 1**). The correlation was calculated between the estimated and ground truth pseudotime of each trajectory. The rest of the parameters were set to their default values (see **Section 3.1**).

Supplementary Figure 4

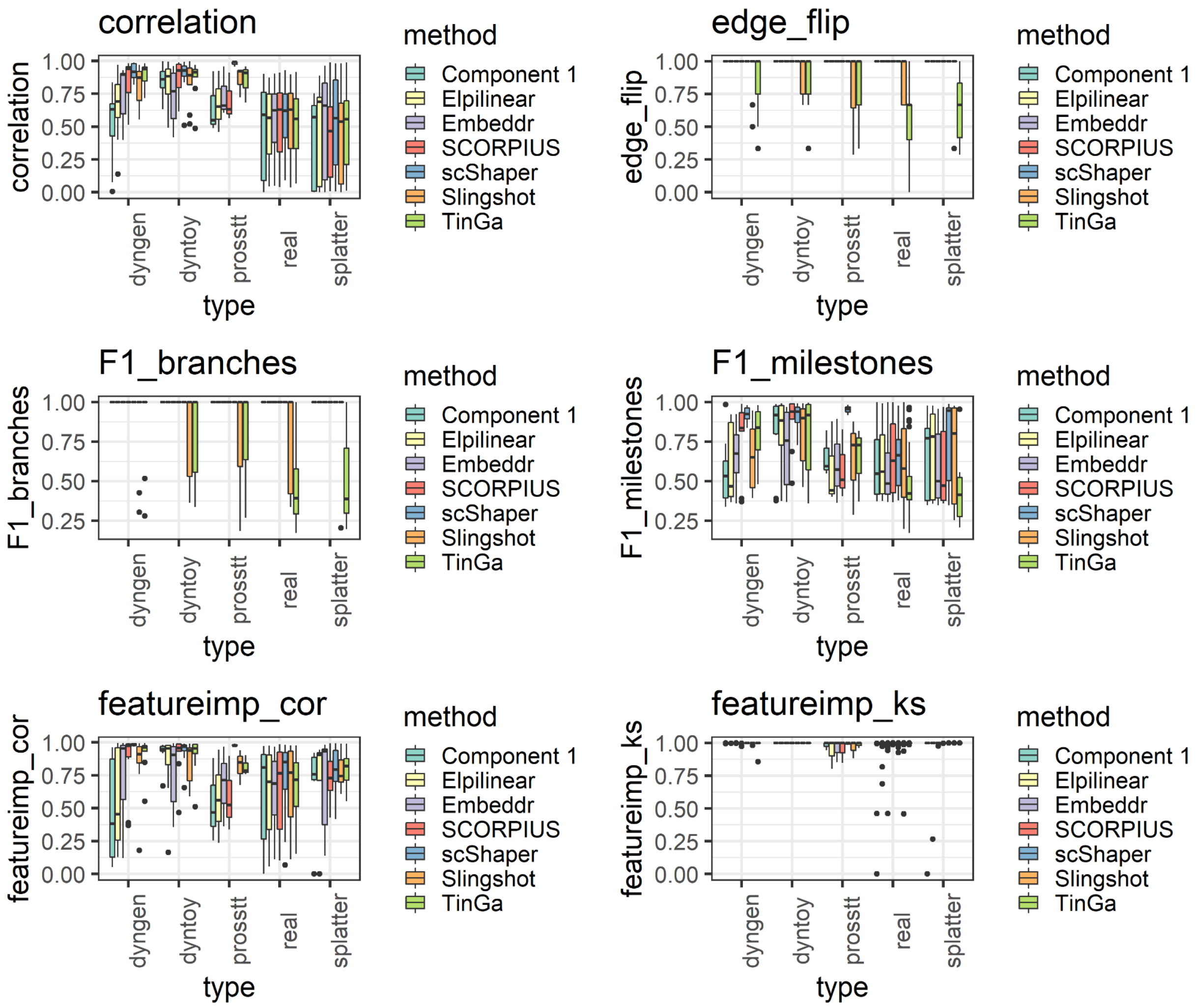

Supplementary Figure 4. The first part of the 17 benchmarking metrics computed using the dynbenchmark framework. See **Supplementary Figs. 5-6** for the visualizations of the rest of the metrics. Descriptions of the metrics are in **Supplementary Table 2**.

Supplementary Figure 5

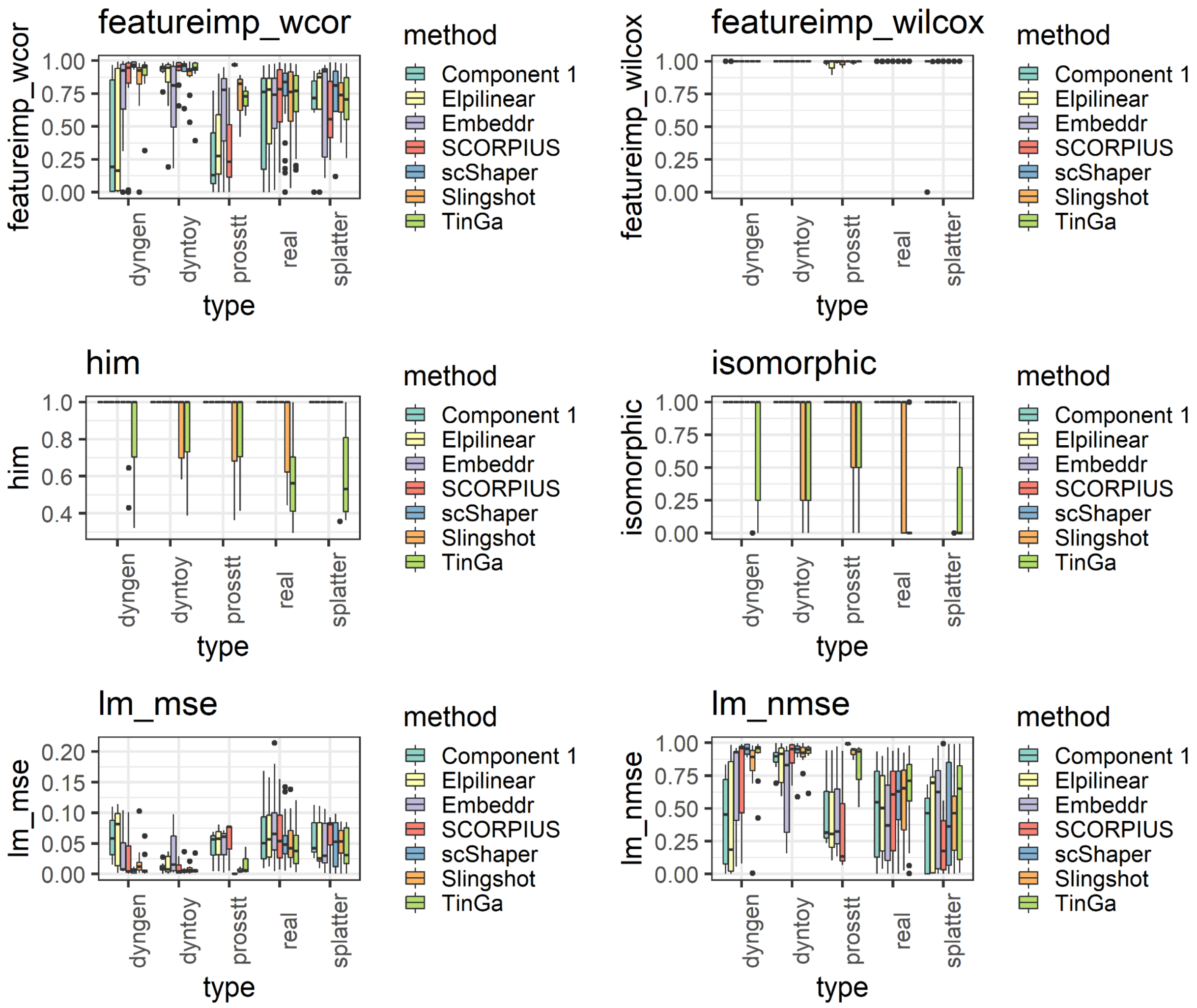

Supplementary Figure 5. The second part of the 17 benchmarking metrics computed using the dynbenchmark framework. See **Supplementary Figs. 4 and 6** for the visualizations of the rest of the metrics. Descriptions of the metrics are in **Supplementary Table 2**.

Supplementary Figure 6

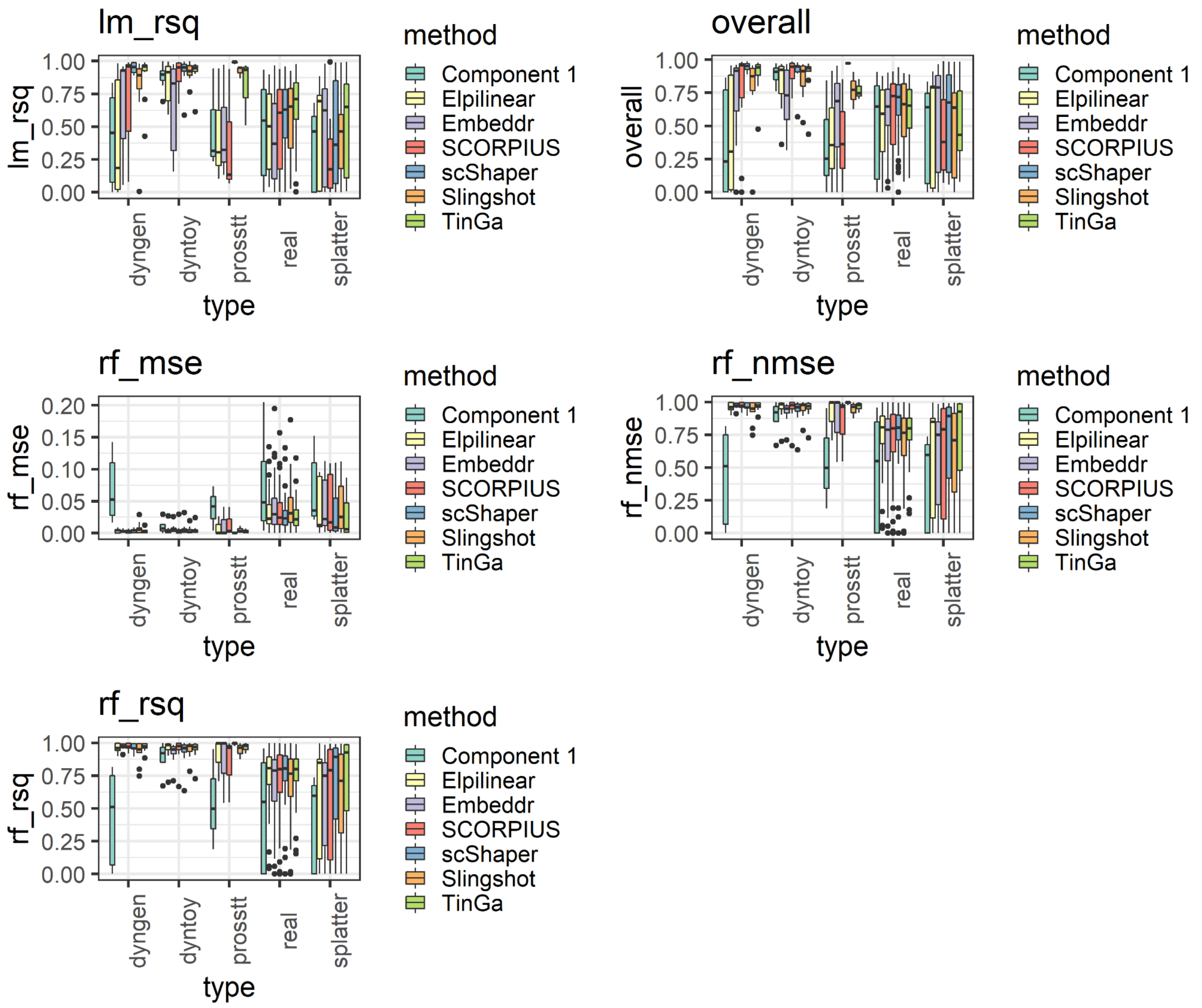

Supplementary Figure 6. The third part the 17 benchmarking metrics computed using the dynbenchmark framework. See **Supplementary Figs. 4-5** for the visualizations of the rest of the metrics. Descriptions of the metrics are in **Supplementary Table 2**.

Supplementary Figure 7

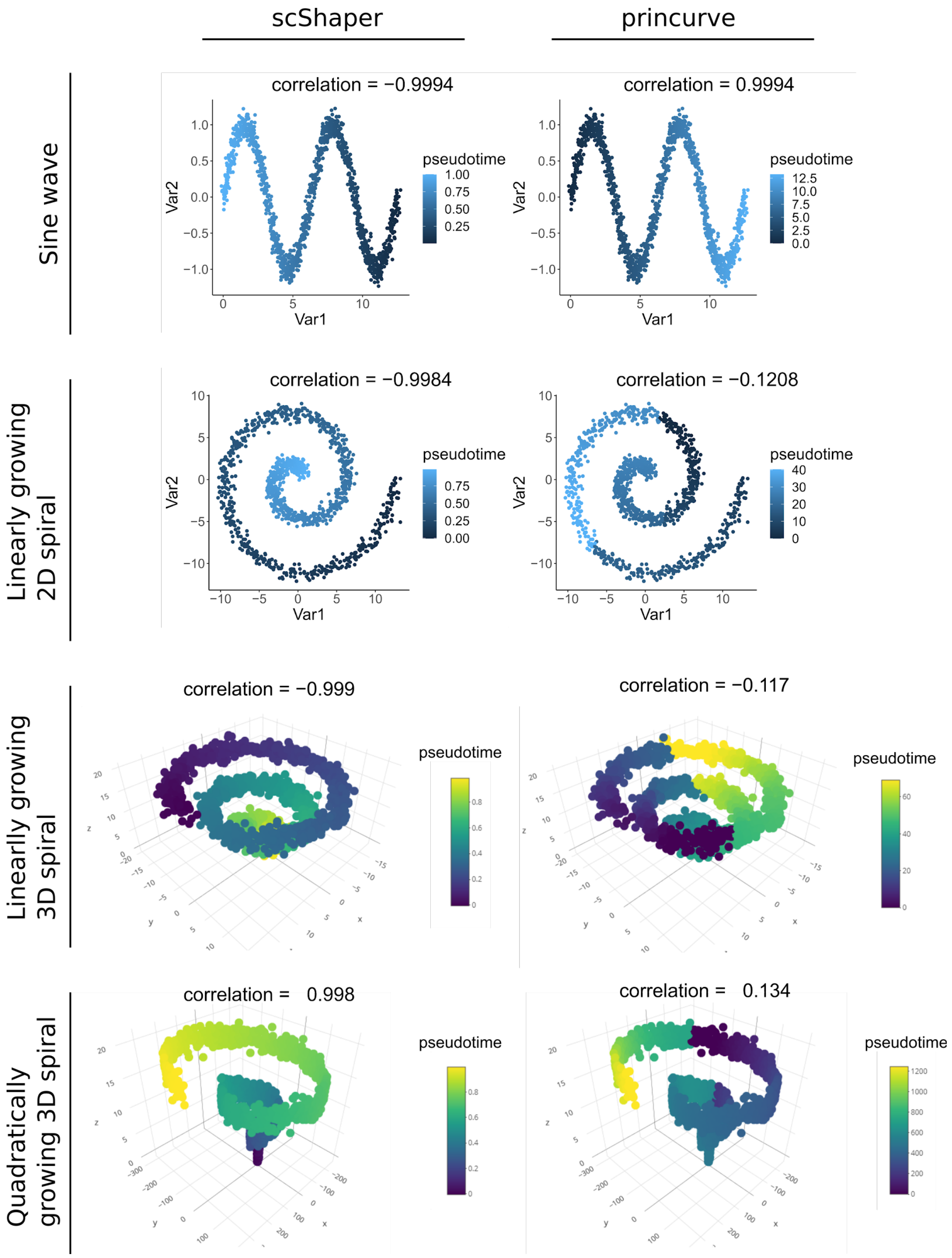

Supplementary Figure 7. Comparison of scShaper and the principal curve method from the princurve R package with data simulated using four trigonometric functions (**Section 2.4 of manuscript**). The correlation value above each plot denotes the Pearson correlation coefficient between the inferred and ground truth pseudotimes.

Supplementary Table 1

Supplementary Table 1. Details of the trigonometric trajectories.

**Name**	**Function**	**Range of *t***
2D spiral	$x = t\ \cos(t), y = t\ \sin(t)$	$[1, 4\pi]$
Quadratically growing 2D spiral	$x = t^2\ \cos(t), y = t^2\ \sin(t)$	$[1, 4\pi]$
3D spiral	$x = t\ \cos(t), y = t\ \sin(t)$, $z = t$	$[1, 6\pi]$
Quadratically growing 3D spiral	$x = t^2\ \cos(t), y = t^2\ \sin(t)$, $z = t$	$[1, 6\pi]$
Sine wave	$x = t, y = \sin(t)$	$[0,\ 4\pi]$

Supplementary Table 2

Supplementary Table 2. Details of the dynbenchmark metrics. Descriptions taken from the documentation of the dyneval R package and https://github.com/dynverse/dynbenchmark_results. The metrics with "time_" prefix were excluded, because they measure the run time for calculating the metrics.

Metric ID	Long name	Category	Perfect score	Worst score	Comment
correlation	Geodesic distance correlation	cell positions	1	0	Correlation between geodesic distances
rf_mse	Random Forest MSE	neighbourhood	0	0.3	Using use the positions of one trajectory to predict the positions within the other trajectory
rf_nmse	Random Forest Normalised MSE	neighbourhood	1	0	
rf_rsq	Random Forest R^2	neighbourhood	1	0	
lm_nmse	Linear regression Normalised MSE	neighbourhood	1	0	
lm_mse	Linear regression MSE	neighbourhood	0	0.3	
lm_rsq	Linear regression R^2	neighbourhood	1	0	
edge_flip	Edge flip	topology	1	0	the minimal number of edges which should be added or removed to convert one network into the other, divided by the total number of edges in both networks
him	Hamming-Ipsen-Mikhailov similarity	topology	1	0	Hamming-Ipsen-Mikhailov distance

isomorphic	isomorphic	topology	1	0	Running the BLISS algorithm to see if two graphs are isomorphic
featureimp_cor	Feature importance correlation	features	1	0	1) Random forest regression to rank all the features according to their importance in predicting the positions of cells in the trajectory 2) Pearson correlation between the rankings of the reference and the prediction.
featureimp_wcor	Feature importance weighted correlation	features	1	0	Same as featureimp_cor, but the feature importance scores in the reference are weighted so that the top features have relatively more impact on the score
featureimp_ks	Feature importance enrichment ks	features	1	0	Kolmogorov-Smirnov for gene set enrichment analysis. Not mentioned in the original comparison by Saelens *et al*.
featureimp_wilcox	Feature importance enrichment wilcox	features	1	0	Wilcoxon test for gene set enrichment analysis. Not mentioned in the original comparison by Saelens *et al*.
F1_branches	Overlap between the branches	branch assignment	1	0	Mapping each cell to its closest branch and performing clustering comparison
F1_milestones	Overlap between the milestones	branch assignment	1	0	Mapping each cell to its closest milestone and performing clustering comparison
overall	Geometric mean of correlation and featureimp_wcor	average	1	0	Unlike in the original comparison by Saelens *et al*., F1_branches and HIM are not included
